# Pathogenic Variant Profile of Hereditary Cancer Syndromes in a Vietnamese Cohort

**DOI:** 10.3389/fonc.2021.789659

**Published:** 2022-01-05

**Authors:** Van Thuan Tran, Sao Trung Nguyen, Xuan Dung Pham, Thanh Hai Phan, Van Chu Nguyen, Huu Thinh Nguyen, Huu Phuc Nguyen, Phuong Thao Thi Doan, Tuan Anh Le, Bao Toan Nguyen, Thanh Xuan Jasmine, Duy Sinh Nguyen, Hong-Dang Luu Nguyen, Ngoc Mai Nguyen, Duy Xuan Do, Vu Uyen Tran, Hue Hanh Thi Nguyen, Minh Phong Le, Yen Nhi Nguyen, Thanh Thuy Thi Do, Dinh Kiet Truong, Hung Sang Tang, Minh-Duy Phan, Hoai-Nghia Nguyen, Hoa Giang, Lan N. Tu

**Affiliations:** ^1^ Ministry of Health, Hanoi, Vietnam; ^2^ University of Medicine and Pharmacy at Ho Chi Minh City, Ho Chi Minh City, Vietnam; ^3^ Oncology Hospital, Ho Chi Minh City, Vietnam; ^4^ MEDIC Medical Center, Ho Chi Minh City, Vietnam; ^5^ Vietnam National Cancer Hospital, Hanoi, Vietnam; ^6^ University Medical Center Ho Chi Minh City, Ho Chi Minh City, Vietnam; ^7^ Cho Ray Hospital, Ho Chi Minh City, Vietnam; ^8^ Department of Oncology, Faculty of Medicine, Nguyen Tat Thanh University, Ho Chi Minh City, Vietnam; ^9^ Medical Genetics Institute, Ho Chi Minh City, Vietnam; ^10^ Gene Solutions, Ho Chi Minh City, Vietnam

**Keywords:** hereditary cancer syndrome, pathogenic variant, genetic carrier screening, carrier frequency, BRCA1

## Abstract

**Background:**

Hereditary cancer syndromes (HCS) are responsible for 5-10% of cancer cases. Genetic testing to identify pathogenic variants associated with cancer predisposition has not been routinely available in Vietnam. Consequently, the prevalence and genetic landscape of HCS remain unknown.

**Methods:**

1165 Vietnamese individuals enrolled in genetic testing at our laboratory in 2020. We performed analysis of germline mutations in 17 high- and moderate- penetrance genes associated with HCS by next generation sequencing.

**Results:**

A total of 41 pathogenic variants in 11 genes were detected in 3.2% individuals. The carrier frequency was 4.2% in people with family or personal history of cancer and 2.6% in those without history. The percentage of mutation carriers for hereditary colorectal cancer syndromes was 1.3% and for hereditary breast and ovarian cancer syndrome was 1.6%. *BRCA1* and *BRCA2* mutations were the most prevalent with the positive rate of 1.3% in the general cohort and 5.1% in breast or ovarian cancer patients. Most of *BRCA1* mutations located at the BRCA C-terminus domains and the top recurrent mutation was NM_007294.3:c.5251C>T (p.Arg1751Ter). One novel variant NM_000038.6(APC):c.6665C>A (p.Pro2222His) was found in a breast cancer patient with a strong family history of cancer. A case study of hereditary cancer syndrome was illustrated to highlight the importance of genetic testing.

**Conclusion:**

This is the first largest analysis of carrier frequency and mutation spectrum of HCS in Vietnam. The findings demonstrate the clinical significance of multigene panel testing to identify carriers and their at-risk relatives for better cancer surveillance and management strategies.

## Introduction

Cancer remains the leading cause of death worldwide with 19.3 million new cases and almost 10 million deaths in 2020 (1). In Vietnam, the number of cases is also on the rise with 188 new cases and 126 cancer-related deaths per every 100,000 people (1). About 5-10% of cancer cases are hereditary and result directly from hereditary cancer syndromes (HCS), a genetic predisposition to cancer due to inherited germline mutations in one or more genes (2, 3). The most common HCS include the hereditary breast and ovarian cancer syndrome (HBOC) and hereditary colorectal cancer syndromes (HCCS). HBOC is caused by germline mutations mainly in the *BRCA1* and *BRCA2* genes; individuals with HBOC tend to have early onset of breast and/or ovarian cancer as well as some other types of cancer (4). HCCS are associated with mutations in various genes and the major types include Lynch syndrome, familial adenomatous polyposis (FAP) and *MUTYH*-associated adenomatous polypopsis (MAP), all of which predispose affected individuals to both colorectal and extracolonic malignancies at an early age (5). Other HCS such as Li-Fraumeni syndrome, Cowden syndrome and Von Hippel-Lindau syndrome all significantly increase risk for a wide spectrum of tumor types.

Identification of individuals with HCS is highly important as it allows for active surveillance, early cancer detection and tailored management strategies. This can be achieved through genetic counselling and testing, which has been routinely available in Western countries. Particularly, recent advances in next-generation sequencing (NGS) technologies have enabled analysis of multiple genes simultaneously, leading to the widespread adoption of multigene panels for hereditary cancer testing in Western healthcare systems (6). However, in Asia including Vietnam, accessibility to such genetic testing service is far limited due to the high cost and lack of trained laboratories and healthcare professionals (7). Consequently, there is currently no information about the incidence rate of HCS, frequency of pathogenic variant carriers and mutation spectrum of HCS-associated genes in the Vietnamese population. This lack of data further creates barrier for public and healthcare providers’ awareness of the importance of genetic testing and risk management for people carrying germline mutations.

Our laboratory used a panel of 17 high- and moderate- penetrance genes recommended by the National Comprehensive Cancer Network (NCCN) guidelines to screen for germline pathogenic mutations associated with HCS. In this paper, we present the results of mutation profile and prevalence of pathogenic mutations from 1165 Vietnamese participants tested in the year of 2020.

## Methods

### Study Group

This study included 1165 individuals across Vietnam who were referred by physicians or self-enrolled in genetic testing at our laboratory from January to December 2020. 403 participants met the referral indications for cancer predisposition assessment in the guidelines of American College of Medical Genetics and Genomics (ACMG) and the National Society of Genetic Counselors (NSGC) (8). In brief, people with personal or family history of 1) a rare cancer (e.g., ovarian, triple negative breast, medullary thyroid), 2) an early onset cancer (e.g., colorectal or breast cancer before the age of 50), 3) two or more different cancers affecting multiple organs or separate locations in the same organ (e.g., bilateral breast cancer); 4) people with family history of multiple relatives on the same side of the family getting the same types of cancer; 5) people who had 10 or more colorectal polyps found during colonoscopies; 6) people with family members previously tested positive for cancer-predisposing mutations, were included in the study. 762 individuals without personal or family cancer history but interested in knowing their mutation carrier status were also recruited. These participants either self-enrolled in the study or were referred by their primary care physicians during annual health checkup or other cancer-unrelated examinations. There was no exclusion criteria. After genetic counseling, all participants approved and gave written informed consent to the anonymous reuse of their genomic data for this study. All genomic data were de-identified and aggregated for the genetic analysis of the cohort. Detailed information about personal and family history of cancer was provided by the referring clinicians or by interview with participants.

### Gene Panel

Our hereditary cancer gene panel consists of 17 high- and moderate- penetrance genes associated with cancer and hereditary cancer syndromes as recommended by the NCCN guidelines (9, 10): *BRCA1, BRCA2, PALB2, PTEN, TP53, CDH1, MLH1, MSH2, MSH6, PMS2, EPCAM, APC, MUTYH, STK11, VHL, RB1, RET.* The list of genes and their association with different types of cancer is provided in **Table S1**.

### Sample Preparation and Sequencing

Each participant provided either 2 mL of peripheral blood or a buccal swab sample. Genomic DNA was extracted from blood samples by the GeneJet whole blood genomic DNA purification minikit (ThermoFisher, USA), or from buccal swab samples by the QIAamp DNA minikit (Qiagen, Germany). DNA fragmentation and library preparation were performed using the NEBNext Ultra II FS DNA library prep kit (New England Biolabs, USA) following the manufacturer’s instructions. Libraries were pooled together and hybridized with predesigned probes for 17 targeted genes (Integrated DNA Technologies, USA). Massive parallel sequencing was performed using NextSeq 500/550 High output kits v2 (150 cycles) on the Illumina NextSeq 550 system (Illumina, USA) with the minimum target coverage of 100x.

### Variant Calling and Analysis

Quality control and data processing were performed as previously described (11). Briefly, the paired-end reads were aligned to human reference genome (GRCh38) using Burrows Wheeler Aligner (BWA) (12). The aligned output was used to compute coverage depth of targeted regions and variant calling was performed using GATK 3.8 (13). Variants were annotated against dbSNP (14), ClinVar (15) and LOVD (16) databases and were analyzed for their molecular consequences using the Ensemble Variant Effect Predictor (17).

Variants were classified according to the classification in the guidelines of The American College of Medical Genetics and Genomics (ACMG) (18). In short, ACMG recommends a five-tier system (“pathogenic”, “likely pathogenic”, variant of uncertain significance”, “likely benign”, “benign”) to determine the pathogenicity using known literature/database and computational predictive programs as main criteria. In this study, we reported both pathogenic and likely pathogenic variants as “pathogenic variants”.

### Sanger Sequencing

Sanger sequencing was performed to confirm all pathogenic variants identified by NGS. First, primers were designed flanking the mutation location using Primer3Plus (19) and synthesized by Integrated DNA Technologies, USA. PCR amplification was prepared using the same genomic DNA samples as above and Q5 High-Fidelity 2X mastermix (New England Biolabs, USA) following the manufacturer’s instructions. PCR products were purified and sequenced by the Genetic Analyzer 3500xl (Applied Biosystems, USA).

## Results

### Participant Demographics

From January to December of 2020, 1165 Vietnamese participants enrolled under two cancer screening programs: a female-oriented screening program (Pinkcare) that had 815 women and a general screening program that had 350 people (244 female, 106 male). The participants either were referred by physcians (77.0%) or self-enrolled (23.0%) in the screening programs. The mean age was 38.8 years old with 95.6% of the participants were between 15 to 64 years old. 403 participants (34.6%) reported to have either family history or personal medical history of cancer. The main type of cancer in those with medical history was breast and/or ovarian cancer. Among 762 participants (65.4%) reported not to have any history of cancer, 595 had their history information provided by the referring physicians, 167 people self-reported their history, which could not be verified. Majority of the participants (96.9%) were unrelated. Participant demographics is provided in **Table 1**.

**Table 1:** Demographics of participants in the study.

		Participants (N = 1165)
Age, mean (SD)		38.8 (± 11.9)
Age group, N (%)		
0-14		27 (2.3)
15-64		1117 (95.6)
≥ 65		21 (1.8)
Gender, N (%)		
All participants	Male	106 (9.1)
	Female	1059 (90.9)
Without Pinkcare	Male	106 (30.3)
	Female	244 (69.7)
Physician referral or self-enrollment, N (%)		
Physician referral		897 (77.0)
Self-enrollment		268 (23.0)
Family and personal history of cancer, N (%)		
Yes	Personal History only	91
	Family History only	279
	Both	33
	Total	403 (34.6)
No	History provided by physician	595
	Self-report	167
	Total	762 (65.4)
Types of cancer in participants with personal history, N		
Breast and/or ovarian		79
Pancreatic		8
Colorectal		7
Uterine		3
Others		29
Relatedness (first and second degree), N (%)		
Yes		36 (3.1)
No		1129 (96.9)

### All Pathogenic Variants

**Figure 1:**
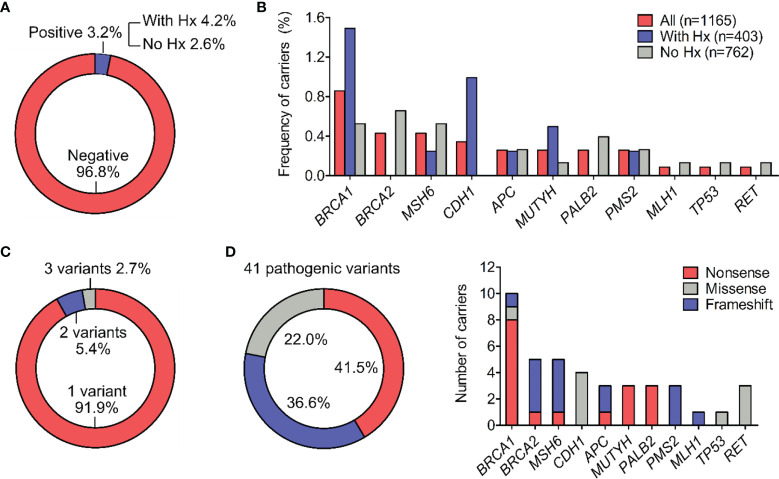
Types and frequency of all pathogenic variants. **(A)** Percentage of participants positive for at least one pathogenic variant in the general cohort and in subgroups with or without history (Hx) of cancer. **(B)** Percentage of pathogenic variant carriers for individual gene. **(C)** Percentage of carriers (n=37) harboring 1, 2 or 3 pathogenic variants. **(D)** Distribution of molecular consequences (nonsense, missense and frameshift) among the 41 pathogenic variants. The frequency of different molecular consequences for each gene was illustrated.

Genetic testing by NGS identified 42 pathogenic variants, 41 of which were confirmed by Sanger sequencing in each individual, demonstrating the accuracy of NGS at 97.6%. 37 out of 1165 participants (3.2%) were positive for at least 1 pathogenic mutation in the gene panel. This frequency among people with family or personal history (Hx) of cancer was 4.2% (17/403) and among those without history was 2.6% (20/762) (**Figure 1A**). Excluding the relatives, the carrier frequency among unrelated people was 2.9% (33/1129).

Out of 17 genes tested, 11 genes had at least 1 mutation while 6 genes: *PTEN, MSH2, EPCAM, STK11, VHL, RB1* showed no mutations. *BRCA1*, *BRCA2* and *MSH6* were the top mutated genes with the carrier frequency of 0.9%, 0.4%, 0.4% respectively among all participants (**Figure 1B** and **Table 2**). The carrier frequency for each gene in the subgroups of people with and without family or personal history is provided in **Table 2**. For example, the percentage of carriers for *BRCA1* mutations was 1.5% in people with history, and 0.5% in those without history (**Figure 1B** and **Table 2**).

**Table 2:** The number and percentage of all pathogenic variant carriers.

	All (n = 1165)		With Hx (n = 403)		No Hx (n = 762)	
	#	%	#	%	#	%
*BRCA1*	10	0.9	6	1.5	4	0.5
*BRCA2*	5	0.4	0	0.0	5	0.7
*MSH6*	5	0.4	1	0.2	4	0.5
*CDH1*	4	0.3	4	1.0	0	0.0
*APC*	3	0.3	1	0.2	2	0.3
*MUTYH*	3	0.3	2	0.5	1	0.1
*PALB2*	3	0.3	0	0.0	3	0.4
*PMS2*	3	0.3	1	0.2	2	0.3
*MLH1*	1	0.1	0	0.0	1	0.1
*TP53*	1	0.1	0	0.0	1	0.1
*RET*	1	0.1	0	0.0	1	0.1

Hx, family or personal history of cancer.

Most of the carriers (91.9%) had one pathogenic variant while 1 person carried 3 pathogenic variants in the *RET* gene and 2 people carried 2 pathogenic variants, one of each in the *CDH1* and *MUTYH* genes (**Figure 1C**). All the variants were found heterozygous.

Nonsense mutations were the most prevalent type, accounting for 41.5% (17/41) of the 41 variants identified in the cohort, followed by frameshift mutations and missense mutations with the frequency of 36.6% (15/41) and 22.0% (9/41) respectively (**Figure 1D**). No insertion/deletion or rearrangement variants were detected. The type of molecular consequences observed was unique to the genes: *BRCA1*, *MUTYH* and *PALB2* mainly had nonsense mutations, *BRCA2*, *MSH6*, *PMS2*, *MLH1* predominantly had frameshift mutations, while *CDH1*, *TP53* had missense mutations (**Figure 1D**). Out of 41 pathogenic variants, 27 of them were unique. The full list of variants, their frequency and molecular consequences is provided in **Table S2**.

### Hereditary Colorectal Cancer Syndromes

**Figure 2:**
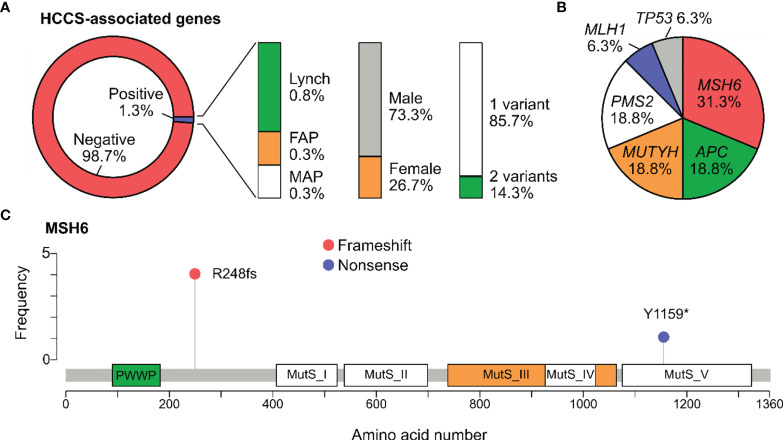
Pathogenic variants associated with Hereditary Colorectal Cancer syndromes. **(A)** Percentage of participants positive for at least one pathogenic variant in the genes associated with Hereditary Colorectal Cancer Syndromes (HCCS): Lynch syndrome (*MLH1*, *MSH2, MSH6, PMS2, EPCAM*), familial adenomatous polyposis (FAP) (*APC*), MUTYH-associated adenomatous polypopsis (MAP) (*MUTYH*). The percentage of carriers by gender and number of variants was also illustrated. **(B)** Pie chart showing the distribution of pathogenic variants among the HCCS-associated genes. No variant was found in *EPCAM* and *MSH2*. **(C)** Lollipop plot reporting distribution of all pathogenic variants identified in *MSH6*. Protein domains shown include PWWP (Pro-Trp-Trp-Pro) and all Mutator S (MutS) domains.

The 3 major types of HCCS include Lynch syndrome (genes: *MLH1*, *MSH2, MSH6, PMS2, EPCAM*), FAP (gene: *APC*), and MAP (gene: *MUTYH*). Frequency of carriers harboring at least one pathogenic variants in the HCCS-associated genes was 1.3% (15/1165). Specifically, the carrier frequency for genes associated with Lynch syndrome, FAP and MAP in the general cohort were 0.8%, 0.3% and 0.3% respectively (**Figure 2A**). The carrier frequency for subgroups of people with and without history is provided in **Table 3**. Majority of the carriers were male (73.3%) and positive for only 1 pathogenic variant (85.7%) (**Figure 2A**). 2 people carried the same 2 pathogenic variants: NM_001048171.1(MUYTH*)*:c.425G>A (p.Trp142Ter) and NM_004360.5(CDH1):c.2195G>A (p.Arg732Gln).

**Table 3:** The percentage of carriers for pathogenic variants associated with specific hereditary cancer syndromes.

		All (n = 1165)	With Hx (n = 403)	No Hx (n = 762)
**HCCS**	All associated genes	1.3%	1.5%	1.0%
Lynch	*MLH1, MSH2, MSH6, PMS2, EPCAM*	0.8%	0.5%	0.9%
FAP	*APC*	0.3%	0.3%	0.3%
MAP	*MUTYH*	0.3%	0.5%	0.1%
**HBOC**	All associated genes	1.6%	1.5%	1.7%
	*BRCA1/2*	1.3%	1.5%	1.2%
	Others *(PALB2, TP53)*	0.3%	0%	0.5%

Hx: family or personal history of cancer.

In total, 16 pathogenic variants were detected in all the HCCS-associated genes except *EPCAM*. *MSH6* was the most frequently mutated gene, accounting for 0.4% of the cases and 31.3% of the HCCS-associated genes (**Figure 2B**). Lollipop plot illustrated the distribution of pathogenic variants along the MSH6 protein sequence, showing that NM_000179.2:c.742del (p.Arg248fs) was the most common mutation and not located in any of the MutS domains of MSH6 (**Figure 2C**).

### Hereditary Breast and Ovarian Cancer Syndrome

**Figure 3:**
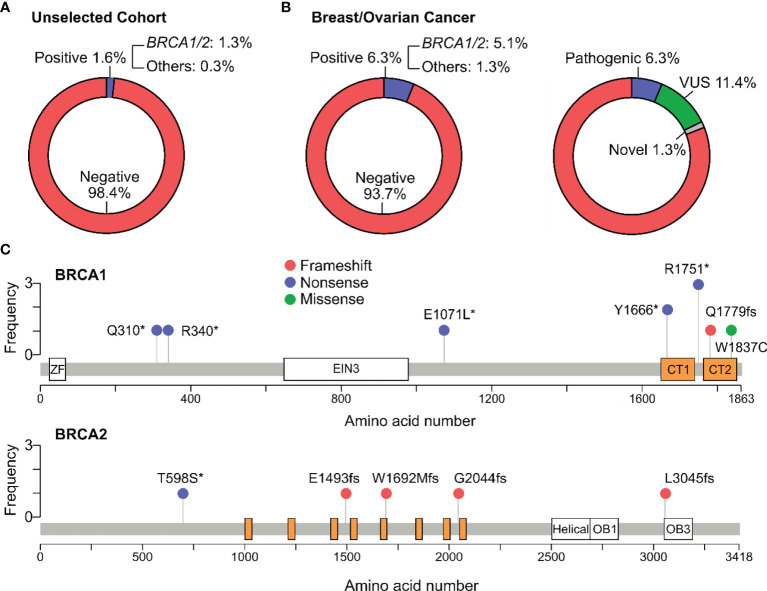
Pathogenic variants associated with Hereditary Breast and Ovarian Cancer syndrome. **(A)** Percentage of participants positive for at least one pathogenic variant in the genes associated with the Hereditary Breast and Ovarian Cancer (HBOC) syndrome: *BRCA1/2* and others: *PALB2, TP53*. No variant was identified in *CDH1*, *STK11* and *PTEN*. **(B)** Percentage of participants with breast or ovarian cancer that harbored pathogenic variants in HBOC-associated genes. Apart from pathogenic variants, variants of uncertain significance (VUS) and a novel variant were identified in these patients. **(C)** Lollipop plot reporting distribution of all pathogenic variants identified in the *BRCA1* and *BRCA2* genes. Protein domains shown in BRCA1 include Zinc finger (ZF), Ethylene insensitive 3 (EIN3), BRCA1 C-Terminus domain 1 (CT1) and domain 2 (CT2). Protein domains shown in BRCA2 include BRC repeats (orange), helical domain, oligonucleotide/oligosaccharide-binding domain 1 (OB1) and domain 3 (OB3).

The carrier frequency for *BRCA1/2* mutations was 1.4% (15/1059) in women and 1.3% (15/1165) in all participants (**Figure 3A**). Apart from *BRCA1/2*, pathogenic mutations were also identified in other genes associated with HBOC (*PALB2* and *TP53*), increasing the prevalence of total carriers to 1.6% (19/1165) (**Figure 3A**). The carrier frequency for subgroups of people with and without history is provided in **Table 3**.

79 participants in our cohort had personal medical history of breast and/or ovarian cancer. The frequency of pathogenic variant carriers among these cancer patients were 6.3%, with *BRCA1/2* carriers accounted for 5.1% (**Figure 3B**). All *BRCA* mutations were identified in the *BRCA1* gene only, not *BRCA2* (**Table S3**). In addition to pathogenic variants, we identified 9 variants of uncertain significance (VUS) in 7 genes and 1 novel missense variant in the *APC* gene in additional 10 cancer patients (**Table S3**). The novel variant: NM_000038.6(APC):c.6665C>A (p.Pro2222His) was the only variant found in a breast cancer patient who had two family members diagnosed with breast cancer and liver cancer. This variant had not been reported in any databases. Analysis by SIFT and Polyphen predicted it to be “deleterious” and “Probably_Damaging” respectively.

Since *BRCA1* and *BRCA2* were the most frequently mutated genes in HBOC, we illustrated the distribution of their pathogenic mutations along the corresponding protein sequences (**Figure 3C**). The most common variant in *BRCA1* was NM_007294.3:c.5251C>T (p.Arg1751Ter) followed by NM_007294.3:c.4997dup (p.Tyr1666Ter). Majority of the variants located at the BRCA C-terminus domains 1 and 2 (CT1 and CT2) of BRCA1. No hotspot was identified for *BRCA2*.

### A Case Study of Hereditary Cancer Syndrome

**Figure 4:**
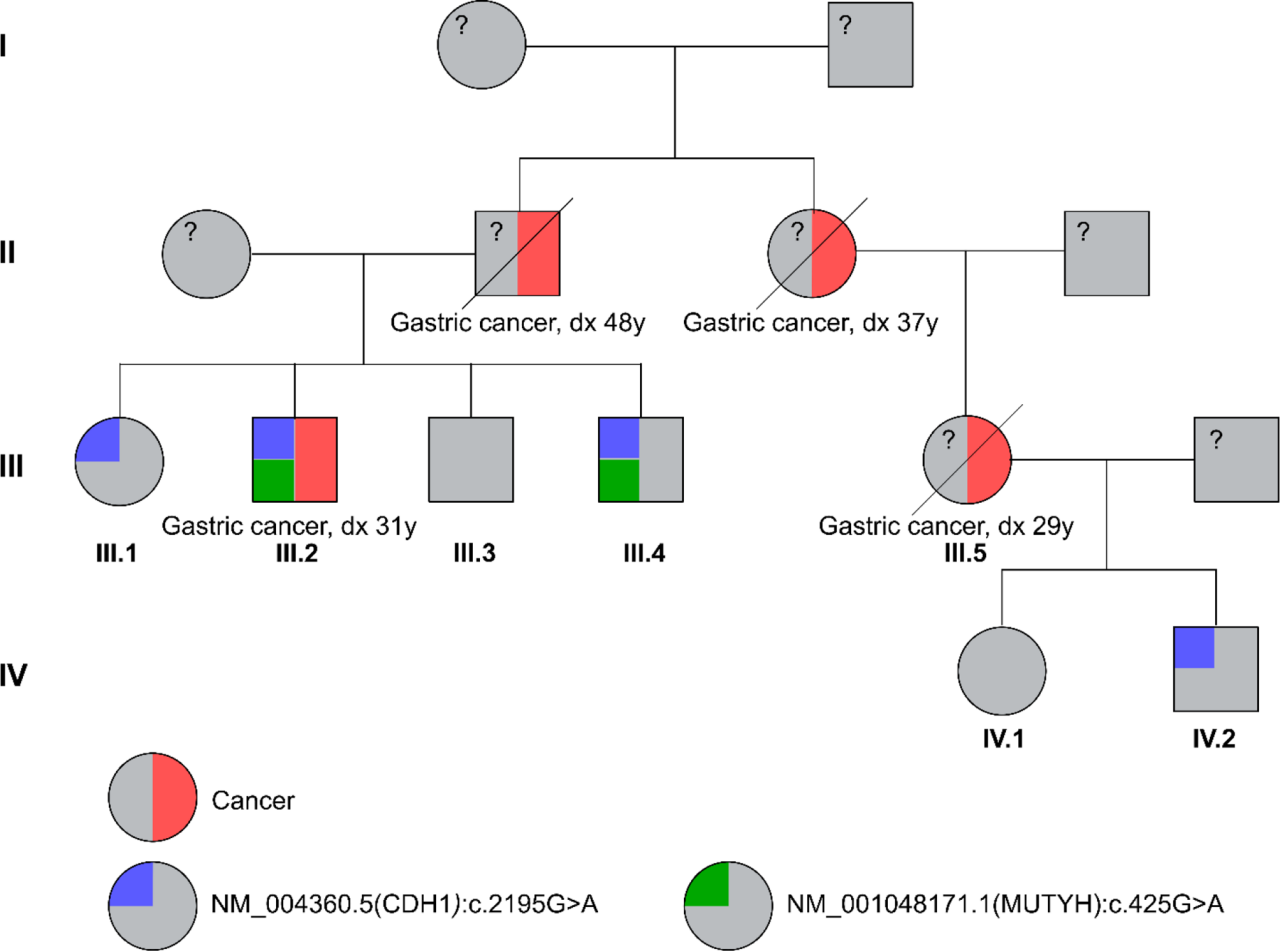
A cancer family pedigree with multiple affected members indicating hereditary cancer syndrome. The proband III.2 was diagnosed with gastric cancer at the age of 31 years old. Genetic testing revealed 2 pathogenic variants in the *CDH1* and *MUTYH* genes. Other family members and relatives were labeled for the presence of cancer and mutation status.

The proband III.2 was diagnosed with gastric cancer at the age of 31 years old. He described a strong family history of cancer, including his father who died of gastric cancer at 48, a paternal aunt who died of gastric cancer at 37 and a paternal first cousin who also died of gatric cancer at 30 (**Figure 4**). Based on his family and medical history of early onset gastric cancer, he was referred to our laboratory for genetic testing for potential hereditary cancer syndrome. Genetic testing using our 17-gene panel revealed 2 pathogenic variants: NM_004360.5(CDH1):c.2195G>A (p.Arg732Gln) and NM_001048171.1(MUTYH):c.425G>A (p.Trp142Ter). His at-risk family members and relatives were then offered genetic counselling and testing for the above mutations by Sanger sequencing. His sister III.1 and the first cousin’s son IV.2 were found positive for the mutation NM_004360.5(CDH1):c.2195G>A (p.Arg732Gln) while his brother III.4 carried the same 2 pathogenic mutations above (**Figure 4**). The mutations were found transmitted through both paternal and maternal lineages and affected several generations, a classic feature of hereditary cancer syndrome. All the affected members received genetic counselling to understand their testing results and got referred to oncologist for cancer risk assessment and management.

## Discussion

Our study used NGS technology to examine the prevalence of carriers for germline HCS-associated variants in 1165 Vietnamese participants. The study cohort was predominantly female (90.9%) because most of them enrolled through our female-oriented screening program Pinkcare. Besides people that met criteria for cancer predisposition assessment (34.6%), majority of the participants (65.4%) did not have any history or risk factors but were interested in knowing their carrier status. The rationale for a mixed cohort was to be able to estimate the frequency of HCS carriers in a more general population, which was found to be 3.2%. This frequency is likely overestimated and not truly reflective of the carrier frequency in the Vietnamese population since the study cohort was still enriched with high-risk individuals and the participants recruited at our laboratory were not representative of the overall Vietnamese population. However, this initial finding helps to raise awareness of HCS in the Vietnamese and serves as a reference for future studies.

*BRCA1* and *BRCA2* were found the most frequently mutated genes with the total prevalence of *BRCA1/2* mutation carriers at 1.3%, or 1 in 78. We compared this finding to a large exome sequencing study from a diverse population-based biobank of 30,223 people. Their subpopulation analysis showed the highest prevalence of carriers in the Ashkenazi Jewish (2%, 1 in 49), followed by the Filipino and other Southeast Asians (1.2%, 1 in 81) (20). Our percentage of *BRCA1/2* mutation carriers at 1.3% in the Vietnamese is similar to other Southeast Asian countries and also to the Han Chinese (1.1%, 1 in 91) (21).

Among 79 participants that had breast and/or ovarian cancer, the total carrier frequency was 6.3% (1 in 16) and for *BRCA1* only was 5.1% (1 in 20); no mutation was identified in *BRCA2*. This frequency of *BRCA* mutation carriers is relatively low compared to 12.6% in Greece, Romania and Turkey (22) and 27.0% in India (23) but comparable to 5.5% in the Han Chinese (21) and 4.7% in the Malaysian (24). However, *BRCA2* mutations were readily reported in all of those populations, suggesting that *BRCA2* mutations might be less prevalent in the Vietnamese patients with breast or ovarian cancers. Interestingly, this conclusion agrees with the observations by Vu et al. who used NGS to characterize all *BRCA* mutations in 101 Vietnamese patients with ovarian cancer. Their data showed that 6.9% (7/101) patients carried pathogenic mutations in *BRCA1* and similar to our study, no *BRCA2* mutation was identified (25). Another study in Vietnam involving 259 breast cancer patients reported only 2 carriers of *BRCA* mutations (0.8%) (26). This extremely low frequency was likely an underestimation because only 17 *BRCA* mutations were tested by Sanger sequencing in their study. It has been demonstrated in several studies that NGS technology allows simultaneous detection of all mutations in multiple genes, hence enabling more accurate estimation of the population incident rate than Sanger sequencing (22, 23, 27).

Majority of *BRCA1* mutations (7/10) located in the BRCA C-Terminus domains, the highly conserved repeats responsible for BRCA1 function. Frameshift or nonsense mutations that disrupt or eliminate the BRCT domains were often reported to increase cancer predisposition (28). Moreover, the mutation landscape of *BRCA1* seems to be unique in our cohort as those reported in the US (29), Greece (22), India (23) and China (30) did not concentrate in any protein domains. This conclusion, however, needs to be corroborated with a larger number of *BRCA1* mutations in future studies. The most prevalent *BRCA1* mutation was NM_007294.3:c.5251C>T (p.Arg1751Ter) as identified in 3 unrelated individuals. This mutation was previously proposed to be a founder mutation in the Vietnamese when it was identified in 4% (4/101) patients with ovarian cancer in Vietnam (25). However, it has also been reported as a founder or recurrent mutation in the Greek (31) and Polish (32), making it a recurrent rather than founder mutation in the Vietnamese.

The percentage of carriers for mutations associated with HCS was 1.3% (1 in 78), specifically with Lynch syndrome: 0.8% (1 in 129), FAP: 0.3% (1 in 388) and MAP: 0.3% (1 in 388). Surprisingly, most of the carriers were male (73.3%) despite the fact that our cohort was female dominant. Most of the studies in the US reported that *MLH1* and *MSH2* mutations were detected in 80-90% cases of Lynch syndrome while *MSH6* and *PMS2* mutations were less than 10% (5, 33). On the contrary, our study found the most mutations in *MSH6* and *PMS2*, only 1 mutation in *MLH1* and no mutation in *MSH2*, similar to a population genetic study in Iceland (34). The variant NM_000179.2(MSH6):c.742del (p.Arg248fs) detected in 4 unrelated individuals is a recurrent mutation in the Vietnamese cohort.

The limitation of this study is that 167 participants who self-enrolled in our screening programs could not have their family or medical history verified. As a result, the subgroup analysis of carrier frequency for people with and without history might be less accurate. Furthermore, our analysis mainly focused on pathogenic variants since they have clear recommendations from the clinical management guidelines. VUS and novel variants that could be specific to the Vietnamese or have clinical relevance are not reported in this study and should be included in more comprehensive mutation profiling in future studies.

In conclusion, our study provides the first insights into the prevalence and mutation spectrum of hereditary cancer syndromes in a large Vietnamese cohort. These findings and the case study of HCS serve as the knowledge base to raise awareness for both the public and healthcare professionals about hereditary cancer syndromes and the importance of genetic counselling and NGS-based genetic testing in Vietnam. The molecular genetic information enables physicians to tailor management plans for inherited cancer patients and engage active surveillance for their at-risk relatives. We anticipate that with the increasing adoption of multigene testing, more comprehensive population-based genetic data and epidemiological information would be available to delineate the role of more genes and variants in cancer predisposition.

## Data Availability Statement

The original contributions presented in the study are included in the article/**Supplementary Material**. Further inquiries can be directed to the corresponding author.

## Ethics Statement

The studies involving human participants were reviewed and approved by the institutional ethics committee of the University of Medicine and Pharmacy, Ho Chi Minh City, Vietnam (approval number 164/HDDD). Written informed consent to participate in this study was provided by the participants or the participants’ legal guardian.

## Author Contributions

VTT, SN, XP, TP, VN, HTN, HPN, PD, TL, BN, TJ, and DN recruited patients and performed clinical analysis. H-DN, NN, DD, VUT, HHN, ML, YN, TD, DT, HT, and M-DP processed samples and analyzed data. HNN, HG, and LT designed experiments, analyzed data and wrote the manuscript. All authors contributed to the article and approved the submitted version.

## Funding

This study was funded by Gene Solutions, Vietnam. The funder did not have any additional role in the study design, data collection and analysis, decision to publish, or preparation of the manuscript.

## Conflict of Interest

H-DN, NN, DD, VUT, HHN, ML, YN, HT, M-DP, HG, and LT are current employees of Gene Solutions, Vietnam.

The remaining authors declare that the research was conducted in the absence of any commercial or financial relationships that could be construed as a potential conflict of interest.

## Publisher’s Note

All claims expressed in this article are solely those of the authors and do not necessarily represent those of their affiliated organizations, or those of the publisher, the editors and the reviewers. Any product that may be evaluated in this article, or claim that may be made by its manufacturer, is not guaranteed or endorsed by the publisher.
