# Supplementary material for: Pathogenic Variant Profile of Hereditary Cancer Syndromes in a Vietnamese Cohort
Source: Front Oncol. 2022 Jan 5;11:789659. doi: 10.3389/fonc.2021.789659 (PMC8767154; doi:10.3389/fonc.2021.789659)
Supplement: Supplementary file 1 [file DataSheet_1.docx]

**Table S1. List of genes in the Hereditary Cancer Gene panel and their associations with various cancer types**

| Gene | Breast | Ovarian | Uterine | Colorectal | Gastric | Pancreatic | Skin | Prostate | Others |
| --- | --- | --- | --- | --- | --- | --- | --- | --- | --- |
| *BRCA1* | ● | ● |  |  |  | ● |  | ● |  |
| *BRCA2* | ● | ● |  |  |  | ● | ● | ● |  |
| *PALB2* | ● | ● |  |  |  | ● |  | ● |  |
| *PTEN* | ● |  | ● | ● |  |  | ● |  | ● |
| *TP53* | ● | ● | ● | ● | ● | ● | ● | ● | ● |
| *CDH1* | ● |  |  | ● | ● |  |  |  |  |
| *STK11* | ● | ● | ● | ● | ● | ● |  |  | ● |
| *MLH1* |  | ● | ● | ● | ● | ● |  | ● | ● |
| *MSH2* |  | ● | ● | ● | ● | ● |  | ● | ● |
| *MSH6* |  | ● | ● | ● | ● | ● |  | ● | ● |
| *PMS2* |  | ● | ● | ● | ● | ● |  | ● | ● |
| *EPCAM* |  | ● | ● | ● | ● | ● |  | ● | ● |
| *APC* |  |  |  | ● | ● | ● |  |  | ● |
| *MUTYH* |  |  |  | ● |  |  |  |  |  |
| *VHL* |  |  |  |  |  | ● |  |  | ● |
| *RB1* |  |  |  |  |  |  |  |  | ● |
| *RET* |  |  |  |  |  |  |  |  | ● |

**Table S2. List of all pathogenic variants identified in the study**

| Gene | Pathogenic Variant | Classification | Frequency |
| --- | --- | --- | --- |
| *APC* | NM_000038.5:c.3927_3931del (p.Glu1309fs) | Frameshift | 0.2% |
| *APC* | NM_000038.6:c.694C>T (p.Arg232Ter) | Nonsense | 0.1% |
| *BRCA1* | NM_007294.3:c.5251C>T (p.Arg1751Ter) | Nonsense | 0.3% |
| *BRCA1* | NM_007294.3:c.4997dup (p.Tyr1666Ter) | Nonsense | 0.2% |
| *BRCA1* | NM_007294.3:c.5511G>T (p.Trp1837Cys) | Missense | 0.1% |
| *BRCA1* | NM_007294.3:c.3214del (p.Glu1071_Leu1072insTer) | Nonsense | 0.1% |
| *BRCA1* | NM_007299.4:c.1018C>T (p.Arg340Ter) | Nonsense | 0.1% |
| *BRCA1* | NM_007294.3:c.5335del (p.Gln1779fs) | Frameshift | 0.1% |
| *BRCA1* | NM_007294.3:c.928C>T (p.Gln310Ter) | Nonsense | 0.1% |
| *BRCA2* | NM_000059.4:c.4478_4481del (p.Glu1493fs) | Frameshift | 0.1% |
| *BRCA2* | NM_000059.3:c.6129del (p.Gly2044fs) | Frameshift | 0.1% |
| *BRCA2* | NM_000059.3:c.5073dupA (p.Trp1692Metfs) | Frameshift | 0.1% |
| *BRCA2* | NM_000059.3:c.9134del (p.Leu3045fs) | Frameshift | 0.1% |
| *BRCA2* | NM_000059.3:c.1796_1800del (p.Thr598_Ser599insTer) | Nonsense | 0.1% |
| *CDH1* | NM_004360.5:c.2195G>A (p.Arg732Gln) | Missense | 0.4% |
| *MLH1* | NC_000003.12:g.37047527_37047540del | Frameshift | 0.1% |
| *MSH6* | NM_000179.2:c.742del (p.Arg248fs) | Frameshift | 0.4% |
| *MSH6* | NM_000179.2:c.3476dup (p.Tyr1159Ter) | Nonsense | 0.1% |
| *MUTYH* | NM_001048171.1:c.425G>A (p.Trp142Ter) | Nonsense | 0.2% |
| *MUTYH* | NM_001128425.1:c.55C>T (p.Arg19Ter) | Nonsense | 0.1% |
| *PALB2* | NM_024675.3:c.2968G>T (p.Glu990Ter) | Nonsense | 0.2% |
| *PALB2* | NM_024675.3:c.2257C>T (p.Arg753Ter) | Nonsense | 0.1% |
| *PMS2* | NM_000535.7:c.746_753del (p.Asp249fs) | Frameshift | 0.3% |
| *RET* | NM_020975.4:c.200G>A (p.Arg67His) | Missense | 0.1% |
| *RET* | NM_020975.4:c.874G>A (p.Val292Met) | Missense | 0.1% |
| *RET* | NM_020975.4:c.2944C>T (p.Arg982Cys) | Missense | 0.1% |
| *TP53* | NM_000546.5:c.799C>T (p.Arg267Trp) | Missense | 0.1% |

**Table S3. List of variants identified in participants with breast or ovarian cancer**

| Pathogenic variants | |
| --- | --- |
| *BRCA1* | NM_007294.3:c.5511G>T (p.Trp1837Cys) |
|  | NM_007294.3:c.4997dup (p.Tyr1666Ter) |
|  | NM_007294.3:c.3214del (p.Glu1071_Leu1072insTer) |
| *MSH6* | NM_000179.2:c.3476dup (p.Tyr1159Ter) |
| Variants of uncertain significance (VUS) | |
| *BRCA2* | NM_000059.3:c.2477A>G (p.Glu826Gly) |
|  | NM_000059.3:c.4109G>C (p.Gly1370Ala) |
| *TP53* | NM_000546.5:c.685T>C (p.Cys229Arg) |
| *MSH6* | NM_000179.2:c.2260A>C (p.Thr754Pro) |
| *PMS2* | NM_000535.7:c.46A>G (p.Lys16Glu) |
| *CDH1* | NM_004360.5:c.1978G>A (p.Val660Met) |
| *STK11* | NM_000455.4:c.*201G>A (p.=) |
| *RET* | NM_020975.4:c.1271A>G (p.Lys424Arg) |
|  | NM_020975.4:c.2234A>T (p.His745Leu) |
| Novel variant | |
| *APC* | NM_000038.6:c.6665C>A (p.Pro2222His) |
